# Prognostic value of tumor-infiltrating lymphocytes for patients with completely resected stage IIIA(N2) non-small cell lung cancer

**DOI:** 10.18632/oncotarget.6979

**Published:** 2016-01-22

**Authors:** Wen Feng, Yuan Li, Lei Shen, Xu-Wei Cai, Zheng-Fei Zhu, Jian-Hua Chang, Jia-Qing Xiang, Ya-Wei Zhang, Hai-Quan Chen, Xiao-Long Fu

**Affiliations:** ^1^ Department of Radiation Oncology, Shanghai Chest Hospital, Shanghai Jiao Tong University, Shanghai, China; ^2^ Department of Radiation Oncology, Fudan University Shanghai Cancer Center, Shanghai, China; ^3^ Department of Pathology, Fudan University Shanghai Cancer Center, Shanghai, China; ^4^ Department of Medical Oncology, Fudan University Shanghai Cancer Center, Shanghai, China; ^5^ Department of Thoracic Surgery, Fudan University Shanghai Cancer Center, Shanghai, China

**Keywords:** lymphocytic infiltration, non-small cell lung cancer, prognosis, survival, tumor-infiltrating lymphocytes

## Abstract

**Background:**

The patient prognosis after complete resection for pathologic stage IIIA(N2) non-small cell lung cancer (NSCLC) remains a significant concern. The clinical relevance of the host immune response to NSCLC has yet to be established. We aimed to investigate the prognostic value of tumor-infiltrating lymphocytes (TILs) in a uniform cohort of patients with completely resected stage IIIA(N2) NSCLC.

**Methods:**

From 2005 to 2012, consecutive patients with pathologic stage IIIA(N2) NSCLC who underwent complete resection at our institution were reviewed. For each case, full-face hematoxylin and eosin-stained sections from surgical specimens were evaluated for the TIL density. A published, recommended TIL scoring scale was followed. The patients were stratified into the TIL− or TIL+ group based on pathologic evaluation.

**Results:**

Data from 320 patients were included in the analysis. Based on a median follow-up duration of 30.8 months, a higher density of TILs was associated with an improved postoperative survival time (*P* = 0.06). Subgroup analyses indicated that this positive effect was the greatest for patients with squamous cell carcinoma (SCC; *P* = 0.03). Among those with SCC, the TIL+ patients experienced a significantly increased 3-year distant metastasis-free survival (DMFS) compared to the TIL− patients (60.6% versus 42.7%, *P* = 0.02). Multivariate analyses of the 93 patients with SCC tumors confirmed that TIL+ was an independent prognostic factor for an increased DMFS (HR = 0.39, 95%CI 0.17–0.87, *P* = 0.02) and a prolonged overall survival (OS; HR = 0.47, 95%CI 0.22–1.00, *P* = 0.05).

**Conclusions:**

Our data suggest a potential role of TILs in predicting the survival of patients with completely resected stage IIIA(N2) NSCLC. The beneficial effects of TILs were more pronounced in the prediction of the DMFS and the OS in patients with SCC. This parameter should be considered for prospective inclusion in clinical trials.

## INTRODUCTION

Stage IIIA(N2) non-small cell lung cancer (NSCLC) consists a heterogeneous group of patients with distinct clinical subsets that can be classified as follows:1) stage IIIA-1/IIIA-2, incidental mediastinal nodal involvement, found either intraoperatively in a single station or in the final pathological examination of the surgical specimen; 2) stage IIIA-3, clinical single station or multistation N2 node(s) involvement documented by computed tomography (CT) and/or positron emission tomography (PET)/CT imaging; and 3) stage IIIA-4, bulky or fixed cN2 involvement identified at imaging [1]. The international guidelines recommend that patients with occult-positive N2 nodes that are discovered at the time of pulmonary resection should continue with the planned resection along with formal mediastinal lymph node dissection and that patients with minimal N2 disease could be considered for a multimodality approach that includes surgical resection [1–3]. In our study, we evaluated patients with occult N2 identified after complete resection (IIIA-1, IIIA-2) and minimal N2 disease identified by CT or PET/CT imaging (IIIA-3). Determination of the role of surgery in a patient with minimal N2 disease was made, prior to the initiation of any therapy, by a multidisciplinary team in our institution. Complete surgical resection is still considered the initial treatment option in our country for most patients with occult or minimal N2 lesions that are determined to be resectable by a multidisciplinary team.

The patient prognosis after complete resection for pathologic stage IIIA(N2) NSCLC remains a significant concern; the 5-year overall survival (OS) rates range from 10% to 30% [4]. Postoperative chemotherapy (POCT) has a modest, but statistically significant, survival benefit for patients with resected NSCLC (absolute improvement in survival of 4% at 5 years) [5]. The postoperative radiotherapy (PORT) meta-analysis [6] described a relative increase in the risk of death with the addition of PORT for completely resected NSCLC. This detrimental effect was evident among patients who had no mediastinal involvement, whereas in patients with stage III and pN2 disease, a slight increase in survival was detected, although the difference was not statistically significant. Growing evidence suggests that PORT that is administered using the modern technique has a favorable effect on the survival of patients with completely resected pN2 disease [7–13]. A large multi-institutional randomized trial evaluating PORT (Lung ART) in this patient population is now under way [14]. Our group has studied the value of PORT and the PORT clinical target volume design for completely resected pathologic stage IIIA(N2) NSCLC [15, 16]. Based on our results, patients with completely resected stage IIIA(N2) NSCLC had a 5-year OS rate of 32.8%; and even after complete resection and POCT, a significant proportion of these patients developed distant metastasis [15, 16]. These findings provided the impetus for identifying patients who are at high risk of recurrence and exploring novel therapeutic approaches for improving patient survival.

There is growing recognition that tumor progression does not simply depend upon its intrinsic behavior but rather involves a complex interaction between the tumor and host antitumor immunity [17, 18]. The effect of the host immune response on tumors has been demonstrated by analyses of *in situ* immune components [19]. Accumulating data have shown that the *in situ* host immune response to a tumor might determine the tumor behavior or provide clinically informative prognostic biomarkers [20–24]. Evidence indicates that the type, density, and location of immune cells within tumors are better predictors of patient survival than the histopathologic methods that are currently used for the staging of colorectal cancer [24]. Promising immunotherapy approaches are under investigation, and an immunological biomarker in the tumor immune microenvironment that could be integrated into future clinical trials and translational research might eventually be identified.

In this regard, we hypothesized that the state of local immune infiltration at the time of NSCLC resection might be clinically important and measureable. Approximately two-thirds of tumor stroma inflammatory cells in NSCLC are lymphocytes; among these lymphocytes, 80% are T cells [25]. The ability of the cellular immune response, as evidenced by tumor-infiltrating lymphocytes (TILs), to predict survival in patients with a variety of solid tumor types has been corroborated by clinical observation [26–29]. However, the impact of the TIL patterns on the disease course of NSCLC remains to be established, and most studies are heterogeneous in terms of the disease stage (I–IV) and TIL assessment method [30–36]. As part of the pathologic workup at the University of Pittsburgh Medical Center, pathologists evaluate resected NSCLC samples for TILs [37, 38]. These pathologists attempted to examine the relationship between the number of TILs and clinical outcome in early-stage NSCLC. Higher TIL levels were associated with improved disease-free survival among patients with resected stage I disease, but these findings did not translate into a survival benefit [37, 38].

In this study, we sought to investigate the prognostic significance of TILs, which were evaluated in routine histopathological sections, in a uniform cohort of patients with pathologic stage IIIA(N2) NSCLC after complete resection.

## RESULTS

### Baseline characteristics

Of the 375 patients who met the inclusion criteria, 18 patients were excluded because they were lost to follow-up. Tumor specimens from 357 patients were evaluated for TILs. For 37 patients, TILs could not be adequately assessed because permanent hematoxylin and eosin (H & E) -stained sections were not available. Therefore, 320 patients were included in the analysis; their characteristics are listed in Table 1. In total, 135 (42%) patients were categorized as TIL+; the remaining 185 patients were defined as TIL−. As shown in Table 1, no significant differences in the clinicopathological characteristics, including gender, age, smoking history, tumor differentiation, angiolymphatic invasion (ALI), number of nodes resected, number of nodes involved, and pathologic T stage, were observed between the two groups. The designation of TIL+ was significantly associated with the histological type (*P* = 0.01). The median numbers of lymph nodes that were resected and involved were 20 (range 3–67) and 4 (range 1–54), respectively, which were adopted as cut-off points. In the entire cohort (*N* = 320), 278 (86.9%) patients received POCT, and 34 (10.6%) patients received PORT. The use of POCT and PORT was well balanced between the two groups. Overall, 99 (73%) patients in the TIL+ group and 140 (76%) patients in the TIL− group received ≥ 4 cycles of POCT (*P* = 0.63), and 18 (13%) and 16 (9%) patients in the TIL+ and TIL−groups, respectively, received PORT (*P* = 0.18).

**Table 1:** Patient characteristics and predictors of the TIL density level

Characteristics	No. of Patients	TIL+ No. (%)	TIL− No. (%)	Sig. (*P* value)
All patients	320	135 (42)	185 (58)	
Gender				0.13
Female	122	58 (48)	64 (52)	
Male	198	77 (39)	121 (61)	
Age (years)				0.45
≤ 60	189	83 (44)	106 (56)	
> 60	131	52 (40)	79 (60)	
Smoking history				0.09
Never/light smoker	155	73 (47)	82 (53)	
Current/heavy smoker	165	62 (38)	103 (62)	
Histological type				0.003
Adenocarcinoma	193	98 (51)	95 (49)	
Squamous cell carcinoma	93	28 (30)	65 (70)	
Adenosquamous carcinoma	23	7 (30)	16 (70)	
Large cell carcinoma	9	1 (11)	8 (89)	
Pleomorphic carcinoma	2	1 (50)	1 (50)	
Histological type				0.01
SCC	93	28 (30)	65 (70)	
Non-SCC	227	107 (47)	120 (53)	
Tumor differentiation				0.44
Well	2	-	2 (100)	
Intermediate	155	64 (41)	91 (59)	
Poor	163	71 (44)	92 (56)	
Angiolymphatic invasion				0.95
ALI−	195	82 (42)	113 (58)	
ALI+	125	53 (42)	72 (58)	
No. of nodes resected				0.41
≤ 20	165	66 (49)	99 (53)	
> 20	155	69 (51)	86 (47)	
No. of nodes involved				0.6
≤ 4	177	77 (57)	100 (54)	
> 4	143	58 (43)	85 (46)	
Pathologic T stage				0.81
T1	73	33 (24)	40 (22)	
T2	210	86 (64)	124 (67)	
T3	37	16 (12)	21 (11)	

Abbreviations: TIL = tumor-infiltrating lymphocyte; SCC = squamous cell carcinoma; ALI = angiolymphatic invasion.

### Prognostic significance of TILs

**Figure 1:**
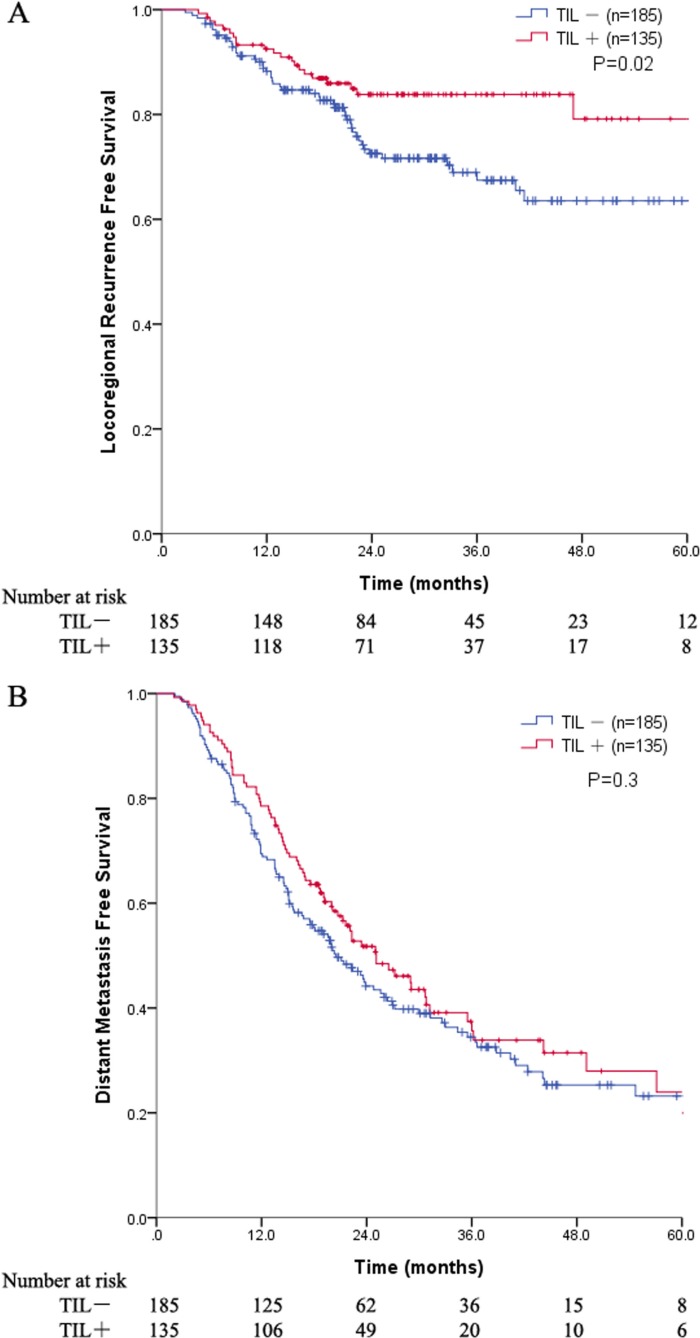
(**A**) Plot of the LRFS for all patients stratified into TIL groups; (**B**) Plot of the DMFS for all patients stratified into TIL groups. Abbreviations: LRFS = locoregional recurrence free survival; DMFS = distant metastasis free survival; TIL = tumor-infiltrating lymphocyte.

**Figure 2:**
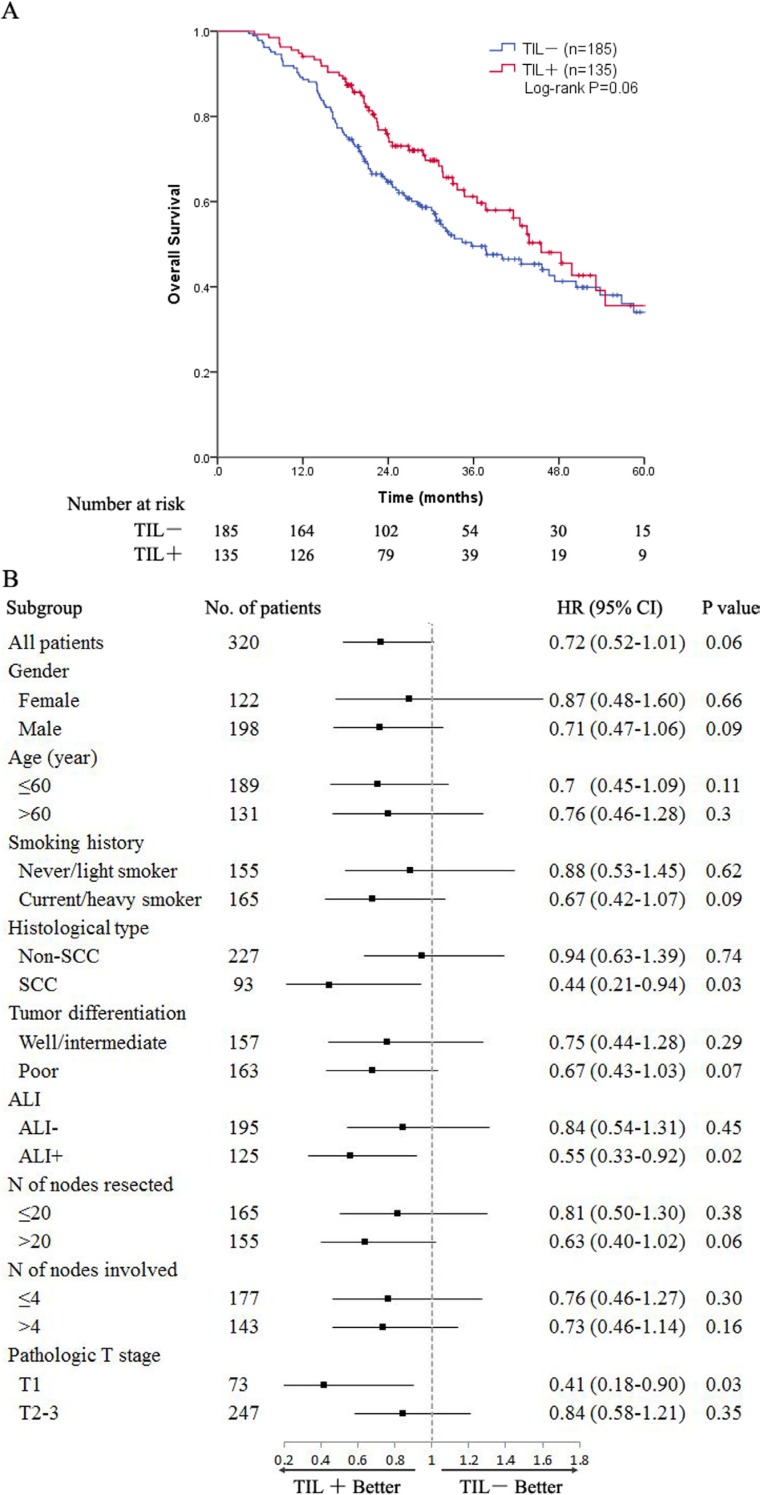
(**A**) Plot of the OS for all patients stratified into TIL groups; (**B**) Subgroup analyses of the OS according to each clinicopathological characteristic. Abbreviations: OS = overall survival; TIL = tumor-infiltrating lymphocyte; SCC = squamouscell carcinoma; ALI = angiolymphatic invasion; CI = confidence interval.

The median follow-up duration was 26.9 months (range, 4.4–101.4 months) for all patients and 30.8 months (range, 12–101.4 months) for living patients. The locoregional recurrence-free survival (LRFS) and the distant metastasis-free survival (DMFS) were compared between the TIL+ and TIL− groups. There were significant differences in the LRFS, favoring the TIL+ group (5-year LRFS 79.2% versus 63.5%, *P* = 0.02) (Figure 1A). However, the 5-year DMFS was 24% for the TIL+ group compared to 23.2% for the TIL− group (*P* = 0.3) (Figure 1B). For all patients, the median survival time (MST) was 42.5 months, and the 1-, 3-, and 5-year OS rates were 90.9%, 54.3%, and 35%, respectively. The survival curves of the four-scale TIL groups are presented in Figure S1. For the patients in the TIL− and TIL+ groups, the MSTs were 35.7 and 45.5 months, respectively. The 1-, 3-, and 5-year OS rates were 88.6%, 49.5%, and 34%, respectively, in the TIL− group and 94.1%, 61.2%, and 35.6%, respectively, in the TIL+ group. The difference in the OS between the two groups trended toward significance (*P* = 0.06) (Figure 2A). In the subgroup analysis, a trend toward a longer OS was observed in all TIL+ subgroups, but significance was only reached for the subgroups of patients with squamous cell carcinoma (SCC; *P* = 0.03) and ALI (*P* = 0.02) as well as pathologic T1 stage (*P* = 0.03) (Figure 2B).

The results of the univariate and multivariate analyses of the relationships between the examined clinicopathological variables and the OS are shown in Table 2. In the multivariate survival analysis, male gender (HR 1.50, 95%CI 1.04-2.15, *P* = 0.03), poor tumor differentiation (HR 1.57, 95%CI 1.12–2.19, *P* = 0.008) and number of involved nodes > 4 (HR 1.72, 95%CI 1.23–2.40, *P* = 0.001) were independently associated with a worse OS. Among the other covariates, the designation of TIL+ was a potential positive prognostic factor (HR 0.7, 95%CI 0.5–0.98, *P* = 0.05).

**Table 2:** Univariate and multivariate analyses of the OS in all patients (*N* = 320)

Characteristics	Univariable analysis			Multivariate analysis	
	MST (m)	5 year (%)	*P* value	HR (95%CI)	*P* value
Gender			0.001		0.03
Female	50.4	44.2%		1	
Male	33.3	29.9%		1.50 (1.04–2.15)	
Age (years)			0.09		0.18
≤ 60	45.5	34.4%		1	
> 60	31.7	37.2%		1.25 (0.90–1.74)	
Smoking history			0.01		0.95
Never/light smoker	49.8	36.4%		1	
Current/heavy smoker	32.5	33.5%		0.99 (0.63–1.55)	
Histological type			0.02		0.14
Non-SCC	49.8	40.3%		1	
SCC	33.3	24.0%		1.31 (0.91–1.88)	
Tumor differentiation			0.001		0.008
Well/Intermediate	53.2	42.9%		1	
Poor	31.3	26.7%		1.57 (1.12–2.19)	
TIL level			0.06		0.05
TIL−	35.7	34.0%		1	
TIL+	45.5	35.6%		0.70 (0.50–0.98)	
ALI			0.02		0.11
ALI−	43.8	42.1%		1	
ALI+	32.1	22.8%		1.31 (0.94–1.82)	
No. of nodes resected			0.66		0.56
≤ 20	41.6	39.2%		1	
> 20	43.8	30.2%		0.91 (0.65–1.26)	
No. of nodes involved			< 0.001		0.001
≤ 4	49.8	47.5%		1	
> 4	31.1	21.9%		1.72 (1.23–2.40)	
Pathologic T stage			0.24		0.83
T1	53.2	42.4%		1	
T2	41.6	35.1%		1.09 (0.71–1.70)	
T3	34.7	22%		1.20 (0.67–2.16)	

Abbreviations: OS = overall survival; MST = median survival time; HR = hazard ratio; CI = confidence interval; TIL = tumor-infiltrating lymphocyte; SCC = squamous cell carcinoma; ALI = angiolymphatic invasion.

### Association of TILs with the histological type

**Figure 3:**
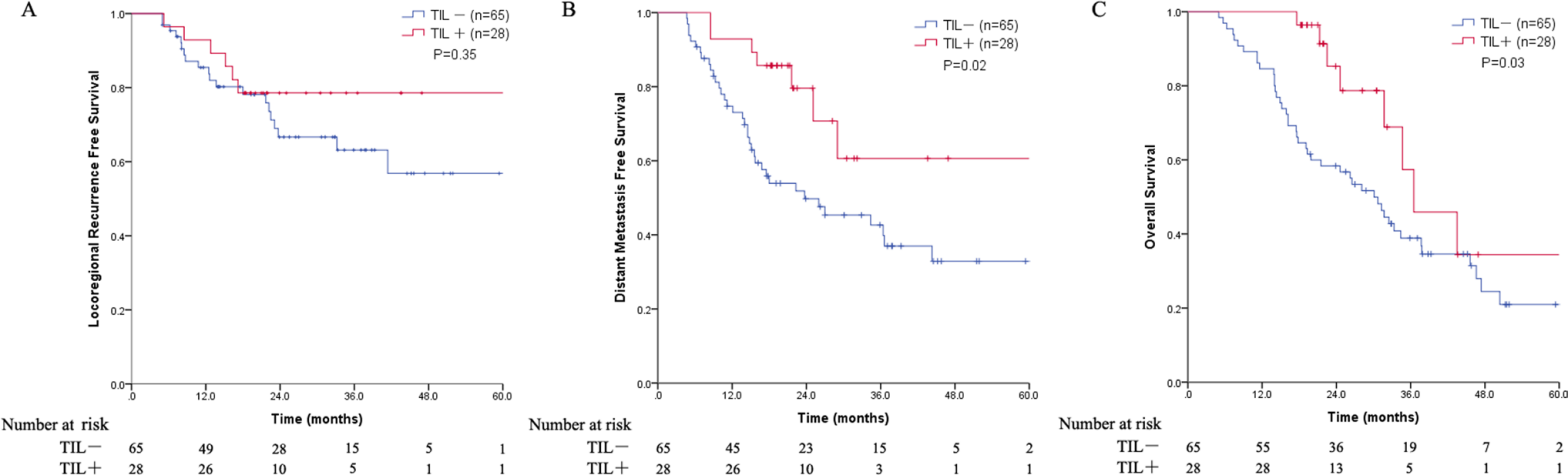
(**A**) Plot of the LRFS for patients with the SCC histological type stratified into TIL groups; (**B**) Plot of the DMFS for patients with the SCC histological type stratified into TIL groups; (**C**) Plot of the OS for patients with the SCC histological type stratified into TIL groups. Abbreviations: LRFS = locoregional recurrence free survival; DMFS = distant metastasis free survival; OS = overall survival; SCC = squamous cell carcinoma; TIL = tumor-infiltrating lymphocyte.

Upon further stratification of the cases based on histological type, for patients with SCC (*N* = 93), the designation of TIL+ was associated with a significantly improved DMFS (3-year DMFS: 60.6% in the TIL+ group versus 42.7% in the TIL− group, *P* = 0.02; Figure 3B). There were no statistically significant differences in the 3-year LRFS between the two groups, although the results regarding the LRFS showed a positive trend in the TIL+ group (*P* = 0.35; Figure 3A). The 1-, 3-, and 5-year OS rates in the TIL+ group were 100%, 57.4%, and 34.4%, respectively, which were significantly higher than the respective OS rates of 84.6%, 38.9%, and 21% in the TIL− group (*P* = 0.03; Figure 3C). The correlation between the TIL level and clinicopathological parameters for the 93 SCC tumors is listed in Table S1. No significant associations were identified between the TIL density and the patient age, gender, smoking history, tumor differentiation, ALI, pathologic T stage, or number of nodes resected and involved (*P* > 0.05). In the multivariate analysis, the designation of TIL+ was a significant independent predictor of improved DMFS (HR 0.39, 95%CI 0.17–0.87, *P* = 0.02). Among the other clinicopathological covariates, TILs had an independent positive prognostic impact on the OS (HR 0.47, 95%CI 0.22–1.00, *P* = 0.05; Table 3).

**Table 3:** Univariate and multivariate analyses of the DMFS and the OS in patients with the squamous cell carcinoma (SCC) histological type (*N* = 93)

Characteristics	*N*	DMFS				OS				
		Univariable analysis		Multivariate analysis		Univariable analysis			Multivariate analysis	
		3 year (%)	*P*	HR (95%CI)	*P*	3 year (%)	5 year (%)	*P*	HR (95%CI)	*P*
Gender			0.25		0.23			0.11		0.25
Female	13	56.2		1		68.4	36.5		1	
Male	80	47.4		1.93 (0.67–5.61)		41.3	22.7		1.73 (0.67–4.47)	
Age (years)			0.35		0.33			0.03		0.05
≤ 60	56	47.5		1		54.9	23.4		1	
> 60	37	54.2		1.37 (0.73–2.56)		29.2	24.3		1.73 (1.00–3.00)	
Smoking history			0.41		0.63			0.34		0.4
Never/light smoker	19	49.7		1		55.4	34.6		1	
Current/heavy smoker	74	48.6		0.76 (0.24–2.37)		42.4	20.4		0.66 (0.25–1.74)	
Tumor differentiation			0.47		0.5			0.79		0.65
Well/Intermediate	45	47.2		1		49.4	24.3		1	
Poor	48	49		0.79 (0.40–1.57)		40.6	23.7		0.86 (0.45–1.65)	
TIL level			0.02		0.02			0.03		0.05
TIL−	65	42.7		1		38.9	21.0		1	
TIL+	28	60.6		0.39 (0.17–0.87)		57.4	34.4		0.47 (0.22–1.00)	
ALI			0.3		0.27			0.3		0.14
ALI−	56	57.6		1		46.1	31.7		1	
ALI+	37	30.2		1.41 (0.76–2.61)		42.8	9.2		1.53 (0.87–2.70)	
No. of nodes resected			0.13		0.22			0.42		0.17
≤ 20	46	43.1		1		43	21.1		1	
> 20	47	54.7		0.68 (0.37–1.26)		46.6	33.2		0.66 (0.36–1.20)	
No. of nodes involved			0.34		0.21					0.16
≤ 4	62	57		1		50.4	27.8	0.16	1	
> 4	31	31		1.52 (0.79–2.93)		33.3	13.3		1.51 (0.86–2.66)	
Pathologic T stage			0.99		0.87			0.7		0.7
T1	15	49.5		1		57.1	21.4		1	
T2	59	53.5		0.91 (0.38–2.16)		41.8	26.6		1.45 (0.62–3.38)	
T3	19	30.7		1.13 (0.39–3.28)		42.5	15.9		1.34 (0.48–3.73)	

Abbreviations: DMFS = distant metastasis free survival; OS = overall survival; TIL = tumor-infiltrating lymphocyte; ALI = angiolymphatic invasion; HR = hazard ratio; CI = confidence interval.

## DISCUSSION

We present a large-scale study that used routine histopathological analysis to investigate the prognostic role of TILs in this population with completely resected pathologic stage IIIA(N2) NSCLC. Overall, our cohort exhibited a better postoperative survival outcome (5-year OS, 35%) than the corresponding historical controls [7–11]. This slightly improved survival might be secondary to more stringent patient selection because a homogeneous group of patients who received complete resection and systematic nodal assessment was selected in our study, providing a relatively uniform clinical background.

We observed a spectrum of TIL staining intensity in the surgically resected stage pN2 NSCLC primary tumor samples, and 42% of which were designated as TIL+ (high TIL infiltration). The proportion of TIL+ patients in our population was consistent with two other studies of early-stage NSCLC [37, 38] that used the same TIL scoring scale, suggesting that TIL infiltration is common in stage IIIA(N2) disease. It was shown that the proportion of TIL+ patients differed between the histological types of NSCLC as follows: TIL+ was more frequent in adenocarcinoma (ADC) patients than in SCC patients, whereas the designation of TIL+ was not associated with the gender, degree of tumor differentiation, or ALI, as has been reported by others [34, 35, 38]. The association between TILs and the histological type was consistent with the observation that in stage III NSCLC, ADC was associated with the highest lymphoid index in the cancer stroma [33]. Another study on NSCLC reported a higher frequency of TIL infiltration within cancer nests in SCC compared to in ADC, but the opposite association was demonstrated for TIL infiltration into the cancer stroma [35]. The difference in the extent of TIL infiltration between the histological types of NSCLC may be due to the heterogeneous tumor antigenicity and/or local environment of each tumor type. However, these findings should be considered and validated in future studies on TILs.

Our data indicated that a high level of TILs in resected NSCLC specimens was associated with improved survival in patients with pathologic stage IIIA(N2) disease after complete resection, suggesting that the majority of patients with a high number of TILs in their primary tumor have had a relatively strong immune reaction against the tumor and, hence, have a better prognosis. This result was consistent with some other studies [26–29, 32, 36–38]. Accumulating data indicate that TIL infiltration in NSCLC is predominantly within the cancer stroma and that the immune response is maximal in the peritumor area [33, 36]. In a study of stage III NSCLC, Lee at al. [33] found that the accumulation of lymphoid cells in the tumor stroma was independently associated with a favorable prognosis. Previously, two studies from the Pittsburgh Medical Center found that a higher level of TILs was associated with improved recurrence-free survival in patients with stage I NSCLC, but these findings did not translate into a significant survival benefit [37, 38]. We reported that a higher density of TILs was associated with an improved LRFS (*P* = 0.02) and that there was a tendency of an improved postoperative survival time in the TIL+ group (*P* = 0.06). This finding of the prognostic significance of TIL+ on local regional tumor control for patients with pN2 disease after complete resection has not been reported in other studies. Actually, the identification of local immunological factors (TIL+) as low-risk prognostic factors for LRF was an important clinical finding that merits and deserves further attention. The lack of a strong association between the degree of TILs and patient survival might reflect the complexity of the *in situ* host immune response to NSCLC. TILs are a heterogeneous group of cells with different functions and additional factors in the tumor microenvironment may regulate the functions of TILs in NSCLC [39]. Moreover, interesting results and further studies in all stages of disease seem warranted.

Given the functional heterogeneity of TILs, it is intriguing that the density of TILs assessed by simple evaluation of H & E-stained primary lung tumor sections has prognostic value in pathologic stage N2 disease despite the lack of detailed information on the TIL subpopulations. These results have several important implications. First, TIL-high NSCLC may define tumors that are more immunogenic, and a study on a single immune subset may have limited value. Low or absent Treg infiltration may indicate that tumors are disregarded by the immune system, while high Treg in tumors may signal an active, but unsuccessful, attempt at tumor rejection. Second, TIL-high and TIL-low NSCLC may each reflect a distinct tumor cell biology that likely has markedly different susceptibility to immunotherapy. Finally, further evidence is needed to assess the clinical importance of the dynamics of TIL subpopulations and to establish an immune score in NSCLC that would have prognostic and predictive value in clinical decision-making.

By further stratifying the cases based on the histological type, we found that the higher OS and DMFS associated with the TIL+ group were statistically significant in the SCC subgroup but not in the ADC subgroup. These findings suggest that the prognostic effect of TILs might differ based on the histological type of NSCLC and indicate that TILs play a unique role in SCC. Similarly, Ruffiniet al. found that the presence of TILs is significantly correlated with improved survival among SCC patients, particularly among those with stage I tumors [34]. A similar result was reported by Al-Shibli and associates, who indicated that stromal CD4+ and CD8+ T lymphocytes were significantly correlated with improved disease-specific survival of NSCLC and that this correlation was limited to SCC [32]. The reasons for this observation are not completely clear. To provide additional evidence supporting the relationship between TILs and histological characteristics, Yoshino and associates examined the phenotypic characteristics of TILs that were freshly isolated from various histological types of lung cancer [40]. They demonstrated differences in the phenotypic characteristics of TILs between ADC and SCC, suggesting that the type of anti-tumor immune response might be different between the histological types of NSCLC. Another finding from a recent study indicated that regulatory T lymphocytes (Tregs), which are known to have host immunosuppressive effects in many solid tumors, are present and significantly enriched in NSCLC patients with ADC compared to those with SCC, which partially confirms that histological characteristics are related to the TIL immune response [41]. We acknowledge that more precise and objective results would be generated if we take into account the high heterogeneity of lung adenocarcinoma and the molecular characterization of this type. Thus, our findings are preliminary, and further study is warranted to confirm them. Notably, the results, which suggest that there are different immunological characteristics between SCC and non-SCC NSCLC patients, have led to further discussions about the response of squamous and non-squamous NSCLC patients to immunotherapeutic agents. Recent clinical data suggest that ipilimumab, administered as first-line treatment, allows for greater improvement for patients in the squamous NSCLC subgroup than for those in the non-squamous NSCLC subgroup [42, 43]. Indeed, additional options for combatting squamous NSCLC are urgently needed [44]. The concept that these immunological characteristics differentially predict susceptibility to immunotherapy and then help individualize adjuvant therapy in the setting of completely resected stage IIIA(N2) NSCLC will be important, clinically relevant questions to address in the near future.

It should be mentioned that our findings are preliminary and that there are limitations to this study. First, given the retrospective nature of this analysis, we cannot prove a direct causal relationship between a high TIL level and prolonged survival. However, this link is highly plausible for the following reason. Although the two populations were not well balanced in terms of the histological type, potential differences in the prognostic factors were corrected in the statistical comparisons. According to the multivariate analysis, the TIL density level was independently associated with an improved OS, even after adjusting for other significant clinicopathological factors, including the histological type. In the SCC subgroup, the correlations between TIL+ and other significant factors were tested, and no significant associations were identified between the density of TILs and other clinicopathological factors. In addition, multivariate analysis was performed to generate further evidence. Second, the semi-quantitative nature of the TIL scoring scale, which is considered to be easy to follow and reproducible in routine pathologic practice, represents a weakness of the present study. There are no currently established thresholds for TILs in NSCLC. The threshold for clinical decisions can be determined once a solid methodology with clinical utility is in place. Therefore, the reproducibility and reliability of this TIL scoring system need to be confirmed in a separate study before it is applied to clinical practice. Third, we focused on the degree of lymphocytic infiltration, which was assessed by the simple evaluation of H & E-stained tumor sections. Given the functional heterogeneity of intratumoral lymphocytes, the analysis of TIL density as a whole might not be sufficient. Thus, further study on the immune subpopulations in the infiltrate is ongoing to establish an immune score that incorporates important immune cell subpopulations. Finally, although our preliminary findings suggested a potential prognostic role of TILs in completely resected stage IIIA(N2) disease and indicated a crucial role of TILs in SCC, they did not provide information about the predictive value of TILs for the therapeutic response. Determining whether the state of the local host immune reaction to the primary NSCLC tumor predicts the therapeutic response, especially the response to immunotherapeutic approaches, is of paramount importance for clinical management, and further investigation is warranted.

In summary, routine histopathological assessment and semi-quantitative scoring of the *in situ* TIL intensity provided clinically meaningful prognostic information for patients with completely resected stage IIIA(N2) NSCLC. The close examination of patients with the SCC histological type revealed that the beneficial effects of TILs were even more pronounced for predicting the DMFS and the OS. Therefore, studies assessing the outcomes and therapeutic efficacies in prospective clinical trials should consider stratifying patients based on the level of TILs.

## MATERIALS AND METHODS

All consecutive patients with pathologic stage IIIA(N2) NSCLC who had undergone complete resection at our institution between January 2005 and June 2012 were retrospectively reviewed. The eligibility criteria for this study were as follows: complete resection through a surgical procedure of either lobectomy or pneumonectomy; systematic node dissection or sampling (a minimum of three N2 stations sampled or complete lymph node dissection) [2]; microscopically tumor-free resection margins; histologically proven stage pT1-3N2M0 NSCLC (according to the TNM classification in the UICC 7th ed. [45]); and postoperative follow-up duration ≥ 4 months. We included patients who received POCT and/or PORT. Patients who underwent wedge resection, received neoadjuvant therapy (chemotherapy and/or radiotherapy), and/or previously presented with malignancies were excluded. Clinicopathological data, including the age, gender, smoking history, histological type, tumor differentiation, ALI, pathologic T stage, and number of lymph nodes resected and involved, were retrieved from the case history and routine pathologic reports. All pathologic characteristics were reported by qualified specialized pathologists at our institution. Smoking history was categorized as never/light ex-smoker (< 100 cigarettes in their lifetime or smoked ≤ 10 pack-years and stopped ≥ 15 years ago) and current/heavy ex-smoker. This study was approved by the institutional review board of our institution.

**Figure 4:**
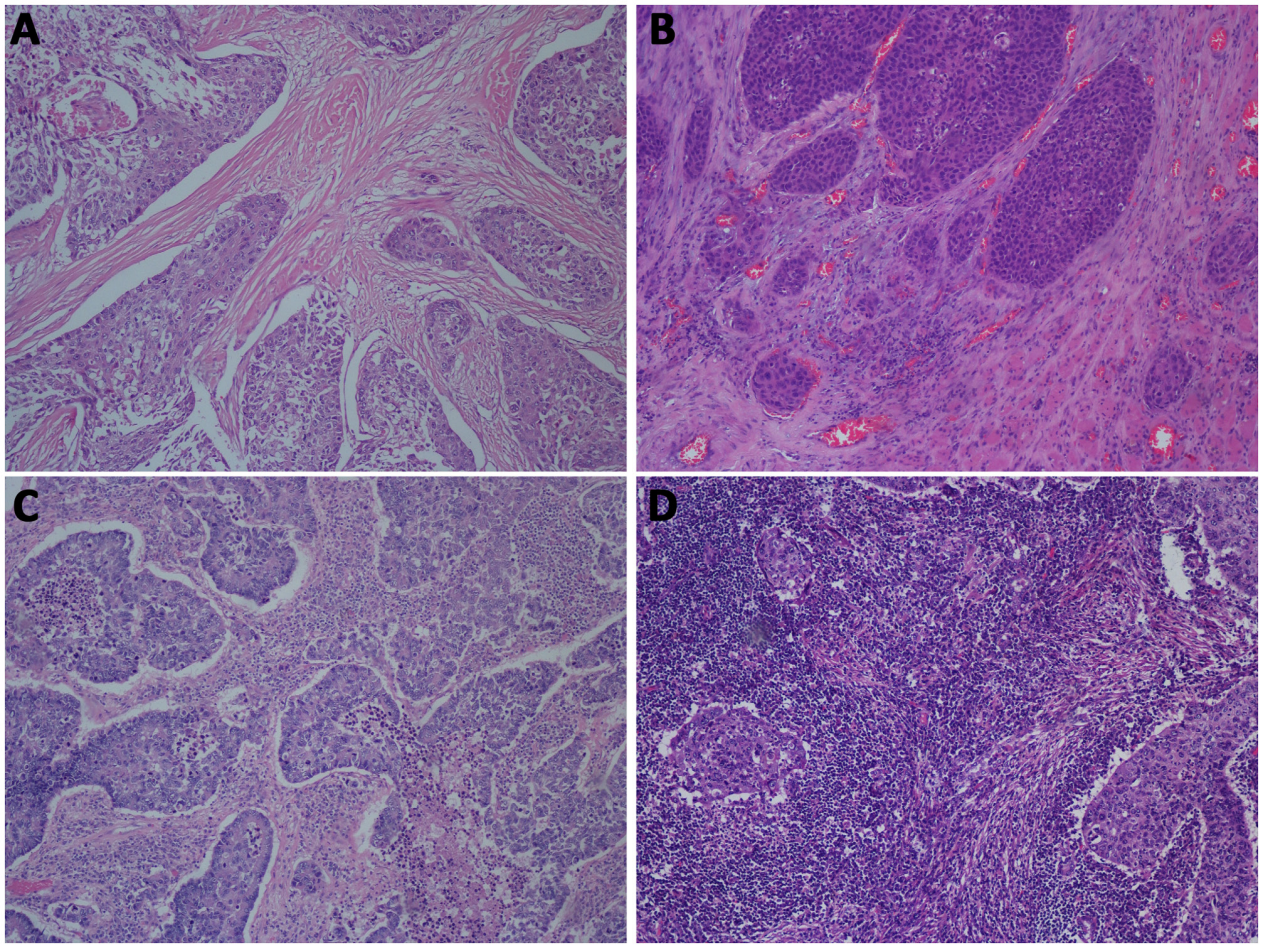
(**A**) TIL− group (score 0): almost no lymphocyte infiltration into the stroma of surrounding cancer nests; (**B**) TIL− group (score 1): low lymphocyte infiltration into the stroma of surrounding cancer nests; (**C**) TIL+ group (score 2): moderate lymphocyte infiltration into the stroma without tumor nest permeation; and (**D**) TIL+ group (score 3): intense lymphocyte infiltration into the stroma and insertion between tumor cells. (100X magnification, H & E-stained sections). Abbreviations: TIL = tumor-infiltrating lymphocyte.

Permanent full-face H & E -stained sections from surgical specimens from each case were retrieved from the pathology archives and evaluated for TILs. The evaluation of TILs was performed according to the TIL scoring scale that was previously described and published by the University of Pittsburgh Medical Center [37, 38]. The degree of lymphocyte infiltration into the tumor was scored as none (score 0), low (score 1), moderate (score 2), or high (score 3) (Figure 4). Low lymphocyte infiltration corresponds to the scattered presence of lymphocytes within the stroma of surrounding cancer nests; moderate lymphocyte infiltration corresponds to the modest presence of lymphocytes in the stroma without tumor nest permeation; and high lymphocyte infiltration denotes an intense or marked presence of lymphocytes in the stroma as well as the insertion of lymphocytes between tumor cells. Patients were stratified into the TIL− (none to low infiltration) or TIL+ group (moderate to high infiltration) based on pathologic evaluation. For each case, the H & E-stained primary lung tumor sections were evaluated for TILs using a magnification of ×100; a minimum of 5 independent fields were evaluated. Those selected fields were considered to be representative of the TIL density in the primary lung tumor block. For evaluating TILs, the boundaries of the invasive tumor were identified and all TILs within the tumor boundaries were scored together as stromal TILs. TILs in areas with crush artifacts, necrosis, and inflammation sites were not scored. In tumors that were heterogeneous in the TIL density in a single tumor section, we evaluated different regions and reported the average level. In cases of disagreement, all sections from the surgical specimen were reexamined to reach a consensus evaluation. Each H & E-stained section was anonymized and independently scored by two expert academic pathologists (L.Y. and S.L.) who were blinded to the clinical characteristics and outcome. A total of 20 randomly selected cases from the total cohort were used to assess the intraobserver and interobserver variation. The Spearman's correlation coefficient between the two pathologists for evaluating TILs was 0.87 (*P* < 0.001).

The patients were generally followed every 3 months after surgery for the first 2 years and every 6–12 months thereafter. The standard follow-up evaluations included clinical assessments, chest CT scans, and ultrasound or CT of the abdomen. Treatment failure was determined by the treating physician based on the available information, including clinical assessments, imaging studies and/or pathology reports. Disease recurrence at the surgical margin, ipsilateral hilum, and/or mediastinum was considered local-regional failure (LRF). All other sites of failure, including the supraclavicular zone, contralateral hilum and distant organs, were considered distant metastasis [46].

Comparisons of the categorical variables between the TIL− and TIL+ groups were performed using the Chi-square test. The OS was defined as the time from surgery to the date of death or date of the last follow-up. The LRFS was defined from the date of surgery to the date of documented LRF or the last follow-up. The DMFS was defined as the time from surgery to the date of distant metastasis or the last follow-up. The LRFS, DMFS and OS probabilities were estimated using the Kaplan-Meier method, and the significance of these results was assessed using the log-rank test. Multivariable Cox proportional hazard models (backward conditional stepwise) were used to adjust for differing risk factor distributions between the groups. Statistical analysis was performed using SPSS (version 20.0, SPSS Inc., Chicago, IL). A value of *P* < 0.05 was considered statistically significant.

## SUPPLEMENTARY MATERIALS FIGURE AND TABLE


