# Supplementary material for: Prognostic value of tumor-infiltrating lymphocytes for patients with completely resected stage IIIA(N2) non-small cell lung cancer
Source: Oncotarget. 2016 Jan 22;7(6):7227–40. doi: 10.18632/oncotarget.6979 (PMC4872781; doi:10.18632/oncotarget.6979)
Supplement: Supplementary file 1 [file oncotarget-07-7227-s001.pdf]

Prognostic value of tumor-infiltrating lymphocytes for patients with completely resected stage IIIA(N2) non-small cell lung cancer

Supplementary Materials

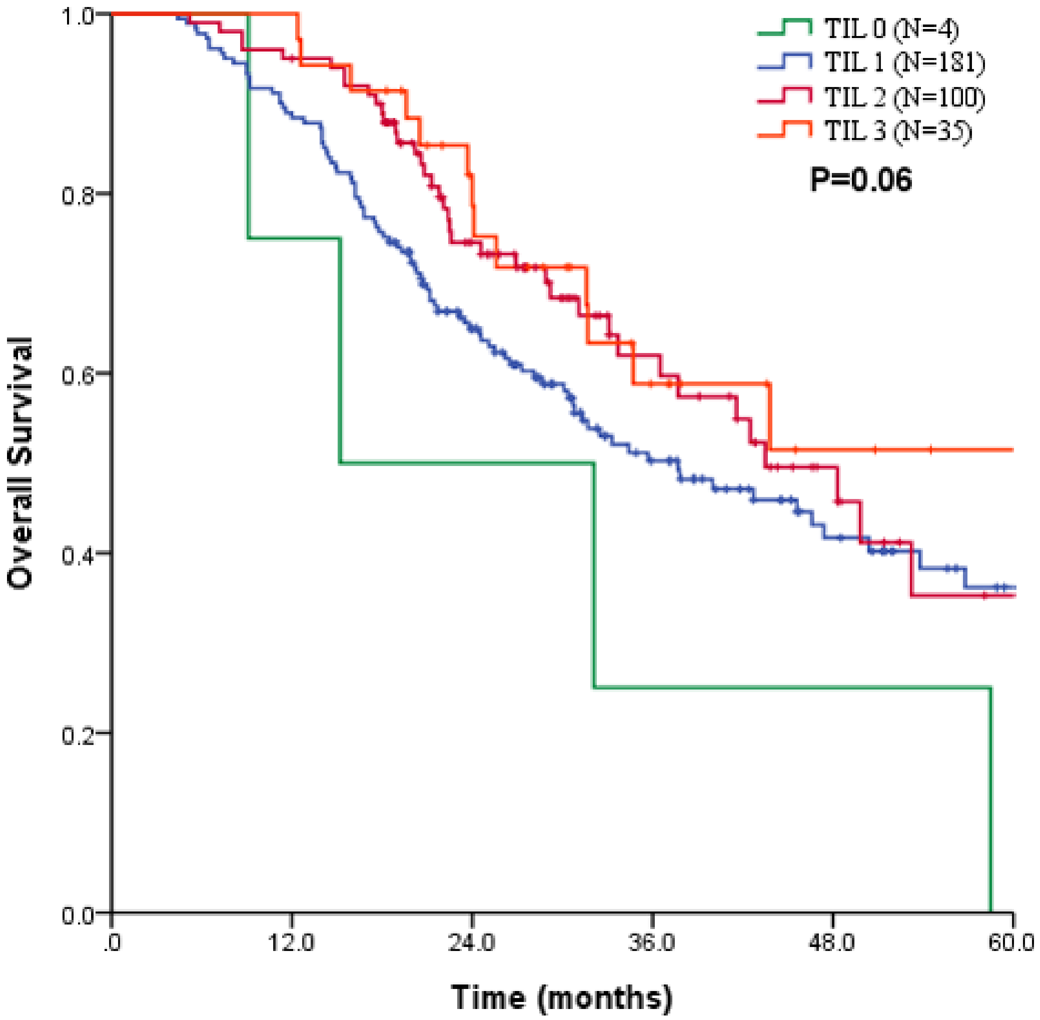

Supplementary Figure S1: Plot of the overall survival for all patients stratified into the four-scale TIL groups.

Supplementary Table S1: Correction of the TIL density level with the clinicopathological characteristics of patients with SCC tumors

Characteristics	TILs level
	***P* value**
Gender, male/female	0.06
Age, ≤ 60/> 60 years	0.15
Smoking history, Never or light smoker/current or heavy smoker	0.47
Tumor differentiation, well or Intermediate/poor	0.81
Angiolymphatic invasion, ALI−/ALI+	0.69
Numbers of lymph node resected, ≤ 20/> 20	0.2
Numbers of lymph node involved, ≤ 4/> 4	0.26
Pathologic T stage, T1/T2/T3	0.18
